# A Negative Feedback Loop in Ultraviolet A-Induced Senescence in Human Dermal Fibroblasts Formed by SPCA1 and MAPK

**DOI:** 10.3389/fcell.2020.597993

**Published:** 2021-06-22

**Authors:** Hongfu Xie, Xiao Xiao, Yuxin Yi, Mingxing Deng, Peihui Li, Dan Jian, Zhili Deng, Ji Li

**Affiliations:** ^1^Department of Dermatology, Xiangya Hospital, Central South University, Changsha, China; ^2^Department of Dermatology, Hunan Provincial People's Hospital, Changsha, China; ^3^Center for Molecular Medicine, Xiangya Hospital, Central South University, Changsha, China; ^4^Key Laboratory of Organ Injury, Aging and Regenerative Medicine of Hunan Province, Changsha, China; ^5^National Clinical Research Center for Geriatric Disorders, Changsha, China; ^6^Science and Technology Aid Program, Xinjiang Uygur Autonomous Region, Urumqi, China; ^7^Hunan Key Laboratory of Aging Biology, Xiangya Hospital, Central South University, Changsha, China

**Keywords:** SPCA1, UVA, intracellular calcium concentration, ROS, MAPK pathway, negative feedback

## Abstract

Secretory pathway calcium ATPase 1 (SPCA1) is a calcium pump localized specifically to the Golgi. Its effects on UVA-induced senescence have never been examined. In our study, expression of SPCA1 was increased in UVA-irradiated human dermal fibroblasts (HDFs) by activating mitogen-activated protein kinase (MAPK) and its downstream transcription factor, c-jun. Dual-luciferase reporter and chromatin immunoprecipitation assays revealed that c-jun regulated SPCA1 by binding to its promoter. Furthermore, downregulating SPCA1 with siRNA transfection aggravated UVA-induced senescence due to an elevation of intracellular calcium concentrations and a subsequent increase in reactive oxygen species (ROS) and MAPK activity. In contrast, overexpression of SPCA1 reduced calcium overload, consequently lowering the ROS level and suppressing MAPK activation. This alleviated the cellular senescence caused by UVA irradiation. These results indicated that SPCA1 might exert a protective effect on UVA-induced senescence in HDFs via forming a negative feedback loop. Specifically, activation of MAPK/c-jun triggered by UVA transcriptionally upregulated SPCA1. In turn, the increased SPCA1 lowered the intracellular Ca^2+^ level, probably through pumping Ca^2+^ into the Golgi, leading to a reduction of ROS, eventually decreasing MAPK activity and diminishing UVA-induced senescence.

## Introduction

Ultraviolet (UV) irradiation is the primary environmental cause of premature skin aging and cell senescence (Bosch et al., 2015). UV radiation can be divided into three parts: UVA (320–400 nm), UVB (280–320 nm), and UVC (200–280 nm). Among three types of UV irradiation, UVA is well-known to be responsible for most of the chronic skin damage associated with cell senescence, due to its abundance and deep penetration into the dermis (Krutmann, 2001). UVA radiation primarily initiates photo-damage through the generation of reactive oxygen species (ROS) (Rinnerthaler et al., 2015). ROS reduce the cellular antioxidant status resulting in oxidative stress, which is one of the most crucial pathogenic factors for cellular senescence.

ROS can directly attack cellular molecules causing telomere shortening, mitochondrial damage, membrane degradation, and oxidation of structural and enzymatic proteins (Yaar and Gilchrest, 2007). More importantly, numerous signal transduction pathways such as mitogen-activated protein kinase (MAPK), nuclear factor-kappa beta/p65, janus kinase, signal transduction and activation of transcription, and nuclear factor erythroid 2-related factor 2 are activated by ROS. Among these, MAPK and its downstream transcription factor, activator protein-1 (AP-1), play crucial roles in cell senescence (Fisher et al., 2002). MAPK pathway is well known to be responsible for the activation of p53 and p16, which are the main causes for cell senescence (Bulavin et al., 1999; Singh et al., 2003). UVA also can upregulate matrix metalloproteinase 1 (MMP1) via the MAPK/AP-1-signaling cascade (Chaiprasongsuk et al., 2017). Moreover, Zheng et al. (2013) reported that 10-hydroxy-2-decenoic acid reduced UVA-induced activation of the c-Jun N-terminal kinase (JNK) and inhibited the expression of MMP-1 and MMP-3, thereby preventing skin photoaging.

SPCA1 is a Golgi-localized transmembrane protein, encoded by the ATPase, Ca^2+^ transporting, type2C, member 1 (ATP2C1) gene, that pumps Ca^2+^ as well as Mn^2+^ into the Golgi apparatus in an ATP-dependent manner (Missiaen et al., 2007; Shull et al., 2011; Praitis et al., 2013). Current studies of SPCA1 are mainly focused on its association with Hailey-Hailey disease (Micaroni et al., 2016; Nellen et al., 2017), secretory cargo sorting (von Blume et al., 2011, 2012), neuron differentiation (Sepulveda et al., 2008, 2009), secretory pathway mammary calcium transport (Reinhardt and Lippolis, 2009), breast cancer (Grice et al., 2010), and focal cerebral ischemia-reperfusion injury (Lehotsky et al., 2009). However, the role of SPCA1 in aging and senescence has never been explored.

One characteristic of SPCA1 attracted our attention. Specifically, SPCA1 expression correlates with oxidative stress and ROS. Pavlikova et al. (2009) demonstrated that ischemic preconditioning could partially suppress lipid and protein oxidation and reverse the depression of SPCA1 induced by ischemia/reperfusion injury in rat hippocampal membranes. Additionally, in neuro-2a cells, SPCA1 knockdown increases the H_2_O_2_-induced production of nitric oxide, 3-nitrotyrosine, and lactate dehydrogenase in a concentration-dependent manner (Fan et al., 2016b). Moreover, SPCA1 plays an important role in cytosolic and intra-Golgi Ca^2+^ homeostasis by transporting Ca^2+^ into the Golgi lumen (Missiaen et al., 2004; Micaroni et al., 2010).

Expression and activity changes of SPCA1 are associated with changes in the intracellular-free Ca^2+^ concentration ([Ca^2+^]i). However, growing evidence indicates a mutual interplay between [Ca^2+^]i and ROS. Abnormally high levels of [Ca^2+^]i induce overproduction of free radicals that can result in oxidative stress. In turn, inordinate accumulation of ROS can exacerbate calcium overload, which further alters ROS production (Gorlach et al., 2015). Because oxidative stress and ROS are the most crucial factors of cell senescence, we speculate that SPCA1 might also play an protected role in skin cell senescence by effecting [Ca^2+^]i and ROS levels.

Therefore, we investigated the role of SPCA1 on UVA-induced cellular senescence and its regulatory mechanism in HDFs.

## Materials and Methods

### Isolation of Dermal Fibroblasts and Cell Culture

Skin samples were collected from healthy male children at 6–12 years of age who were circumcised in the Department of Urology, Xiangya Hospital, Central South University. Primary HDFs were obtained by explantation from samples. The cells were grown in complete Dulbecco's modified Eagle's medium (Gibco, Grand Island, NY, USA) containing 10% fetal bovine serum (Gibco) and antibiotics (penicillin, 100 U/ml; streptomycin, 100 μg/ml) at 37°C in a humidified incubator with 5% CO_2_. Medium was refreshed every 2–3 days, and fibroblasts were split 1:2–1:3 when they reached 80–90% confluence. For all experiments, cells were used at passages 4–6. Before harvesting primary HDFs, written informed consent was obtained from legal guardians of donors in accordance with a protocol approved by the Clinical Research Ethics Committee at the Xiang Ya Hospital of Central South University in Changsha, China.

### UVA Irradiation

Irradiation was carried out using a UVA phototherapy instrument (SS-03A, Sigma, Shanghai, China). After two washes with phosphate-buffered saline (PBS), cells were incubated in PBS under UVA irradiation. Cells were irradiated with a UVA dose of 10 J/cm^2^/day for 3 days. The time interval of these three UVA irradiations is 24 h. After each UVA exposure, cells were fed fresh complete culture medium with or without 10 μM SP600125 (#S1876; Beyotime) or 10 μM SB203580 (#S1863; Beyotime) for 24 h before being collected for further analysis.

### Western Blots

Proteins were extracted from cultured cells by homogenization on ice in radio immunoprecipitation assay lysis buffer (Beyotime, Haimen, China) containing Protease Inhibitor Cocktail (Sigma-Aldrich, St. Louis, MO, USA). Supernatants were obtained after centrifugation at 12,000 × g at 4°C for 10 min. Protein concentrations were determined using a Pierce BCA Protein Assay Kit (ThermoFisher Scientific, Waltham, MA, USA). The remainder of the lysates was mixed with 5 × sodium dodecyl sulfate (SDS) loading buffer (Dingguo, Beijing, China) at a ratio of 1:4. Protein samples were heated at 100°C for 5 min and separated by SDS-polyacrylamide gel electrophoresis. The separated proteins were then transferred to a polyvinylidene difluoride membrane and blocked in 1 × PBST containing 5% (*w*/*v*) skim milk. The membrane blots were incubated overnight at 4°C with primary antibodies.

The primary antibody for ATP2C1 was purchased from Proteintech (Rosemont, IL, USA), and the primary antibody for P16INK4A was purchased from Boster (Wuhan, China). Primary antibodies for phospho-p38, p38, phospho-JNK, JNK, phospho-ERK1/2, and ERK1/2 were purchased from Cell Signaling Technology (Danvers, MA, USA). All antibodies were diluted in 1 × PBST containing 5% BSA and 0.02% sodium azide and were then incubated overnight at 4°C. This was followed by incubation with horseradish peroxidase-conjugated secondary antibodies (Sigma-Aldrich) for 1 h at room temperature. Each membrane was washed in 1 × PBST and developed using a ChemiDo MP System (Bio-Rad, Hercules, CA, USA). The chemiluminescence signal was detected using Image Lab software (Bio-Rad). Protein levels were first normalized to β-actin (Bioworld Technology, St. Louis Park, MN, USA) and then to experimental controls.

### Quantitative Real-Time Polymerase Chain Reaction

Cells were lysed in Trizol (Invitrogen, Carlsbad, CA, USA), and the homogenate was separated into aqueous and organic phases by adding bromochloropropane. Next, total RNA was precipitated from the aqueous phase with isopropanol, and finally washed with ethanol and solubilized in diethyl pyrocarbonate. cDNA was synthesized by reverse transcription from 3 μg of total RNA using the RevertAid First-Strand cDNA Synthesis Kit (Fermentas, Burlington, ON, Canada). The cDNA was diluted 10:1 and amplified using specific primers for ATP2C1 or GAPDH (purchased from Sangon Biotech, Shanghai, China). The following primer pairs were used: 5′-GTA AAA TAC TGC AAC CTT TGG-3′ and 5′-GGT GTG AAA GAA GCT GTT ACA AC-3′ for ATP2C1; 5′-CATTGACCTCAACTACATGGTTTAC-3′ and 5′-GTGATGGGATTTCCATTGATGAC-3′ for GAPDH. Signal detection was performed in triplicate using CFX Manager Software (Bio-Rad). The reaction was performed with initial denaturation at 95°C for 10 min, followed by 40 PCR cycles of 95°C for 15 s and 60°C for 60 s. Data were collected and analyzed using the 2^ΔΔCt^ method. Values of genes were first normalized against GAPDH, and then compared with the experimental controls.

### Small Interfering RNA Transfection

HDFs were transfected with SPCA1 siRNA using Lipofectamine 2000 Transfection Reagent (Thermo Fisher Scientific). The transfection mix (100 pmol RNA and 5 μl Lipofectamine 2000), each diluted in 500 μl Opti-MEM per 60-mm dish was added followed by a 20-min incubation at room temperature. The medium was changed after 6 h. Subsequent operations were done after 48-h incubation. siRNA against SPCA1 targeting the sequence 5′-AAGGTTGCACGTTTTCAAAAA-3′ in the SPCA1 cDNA was purchased from GenePharma (Shanghai, China). The level of knockdown of SPCA1 expression was determined by Western blot.

### Senescence-Associated β-Galactosidase (SA-β-gal) Staining

The Senescence-Associated β-Galactosidase Staining Kit (Cell Signaling Technology) was used according to the manufacturer's instructions. Stained cells were observed under an inverted microscope for the development of blue color. The population of SA-β-gal-positive cells was determined by counting 10 microscopic fields per dish randomly, and then computing an average. The proportions of cells positive for SA-β-gal activity are given as percentages of the total number of cells counted in every dish.

### 3-(4,5-Dimethylthiazol-2-yl)-2,5-Diphenyltetrazolium Bromide Assay

SPCA1-siRNA, SPCA1-cDNA, and control (NS-siRNA, SPCA1-vector)-transfected HDF cells were planted on 96-well plates at an density of 4,000 cells per well in triplicate and exposed to UVA or not. After additional incubation for 0, 24, 48, or 72 h, 20 ml of 3-(4,5-dimethylthiazol-2-yl)-2,5-diphenyltetrazolium bromide (MTT) stock solution (5 mg/ml MTT reagent diluted in PBS; Sigma-Aldrich, USA) was added to each well. The plates were further incubated for 4 h at 37°C and 5% CO_2_ in the dark. The supernatant was carefully removed without disturbing the sediment, and 150 μl dimethyl sulfoxide (Sigma-Aldrich, USA) was added to the wells to dissolve the purple formazan crystals. The absorbance at 490 nm was obtained from a microplate reader (BioRad).

### [Ca^2+^]i Measurements

HDFs were loaded with 4 μM Fluo-4/AM (Dojindo Laboratories, Kumamoto, Japan) for 60 min at 37°C in the dark and washed thrice with PBS. Then, they were digested by trypsin, centrifuged at 2,000 rpm for 5 min, washed two times with PBS, and incubated for another 30 min. Fluorescence was measured using a flow cytometer.

### ROS Measurements

The intracellular ROS level was measured using a Reactive Oxygen Test Kit (Beyotime). After three times UVA exposure, HDFs were fed fresh complete culture medium for 24 h before ROS measurements. HDFs were loaded with dichlorofluorescin diacetate for 20 min at 37°C in the dark and washed thrice with PBS. Fluorescence was measured using a Multiskan Spectrum microplate reader (Thermo Fisher Scientific).

### Plasmids

The SPCA1 promoter reporter [SPCA1-luc (luciferase)] was prepared by inserting an approximately 1.0 kb upstream sequence (based on the putative translation starting codon) into the pGL3-basic vector. This fragment was generated by PCR with 5′-CGGGGTACCAAGTGGTTCTGCAGTAT-3′ and 5′-CCCAAGCTTATATTAGCTAGCTGGTGACTT-3′ as the primers (Sangon Biotech).The SPCA1 overexpression plasmid was prepared by inserting a coding sequence into pcDNA3.1 (+). This fragment was generated by PCR with the following primers: 5′-CCCAAGCTTATGAAGGTTGCACGTT-3′ and 5′-CGGGGTACCTCATACTTCAAGAAAAGATG-3′ (Sangon Biotech). The pGL3-basic and PLR-TK vectors were purchased from Promega. pcDNA3.1 (+) was purchased from Thermo Fisher Scientific. p6600 MSCV-IP N-HA only JUN, pLX304-FOS-V5 were purchased from addgene (Cambridge, MA, USA). All constructs were subjected to sequence analysis.

### Dual-Luciferase Reporter Gene Assay

HEK293T cells were cultured in DMEM (Gibco) containing 10% fetal bovine serum (Gibco) to approximately 60% confluence in a 96-well plate, then co-transfected with different DNA mixes for 24–36 h. Firefly and Renilla luciferase activities were measured using the Dual-Luciferase Reporter Assay System (Promega). Renilla luciferase activity was normalized to firefly luciferase activity. Two thousand base pairs (bp) before transcription initiation sites within the DNMT1 promoter were cloned and inserted into a pGL4 control vector (Promega). Additionally, mutant reporter genes were created using the QuikChange Lightning Multi Site-Directed Mutagenesis kit (Stratagene, La Jolla, CA, USA). The primer used to clone the DNMT1 promoter was following: 5′-GCCGGTACCAAGTGGTTCTGCAGTATACAG-3′ and 5′-GCCCTCGAGTATTAGCTAGCTGGTGACTT-3′ (Sangon Biotech).

### Chromatin Immunoprecipitation Assay

The chromatin immunoprecipitation (ChIP) assay was performed according to the manufacturer's manual using an EZ ChIP kit purchased from Millipore (Temecula, CA, USA). The immunoprecipitated complexes were incubated at 4°C overnight with the indicated antibodies. Bound DNA fragments were analyzed by RT-PCR using the HotStart Taq enzyme (Takara, Dalian, China). Primers were specific for the predicted binding sites (Supplementary Table 2). GAPDH was used as the negative control.

### Nucleofector for Fibroblasts

Cultured primary HDFs (1 × 10^6^) were transfected with the 4D-Nucleofector System (Lonza, Walkersville, MD, USA) according to manufacturer's instructions using the program U-023 preset with 2 μg plasmid DNA or 2 μg pmax GFP vector.

### Statistical Analyses

Data are shown as means ± SD of at least three independent experiments. Statistical significance was assessed using Student's *t*-test or two-way ANOVA with SPSS 17.0 (IBM, Chicago, IL, USA).We considered *P* < 0.05 to be statistically significant.

## Results

### UVA Irradiation Increases the Expression of SPCA1 by Activating the MAPK Pathway

**Figure 1:**
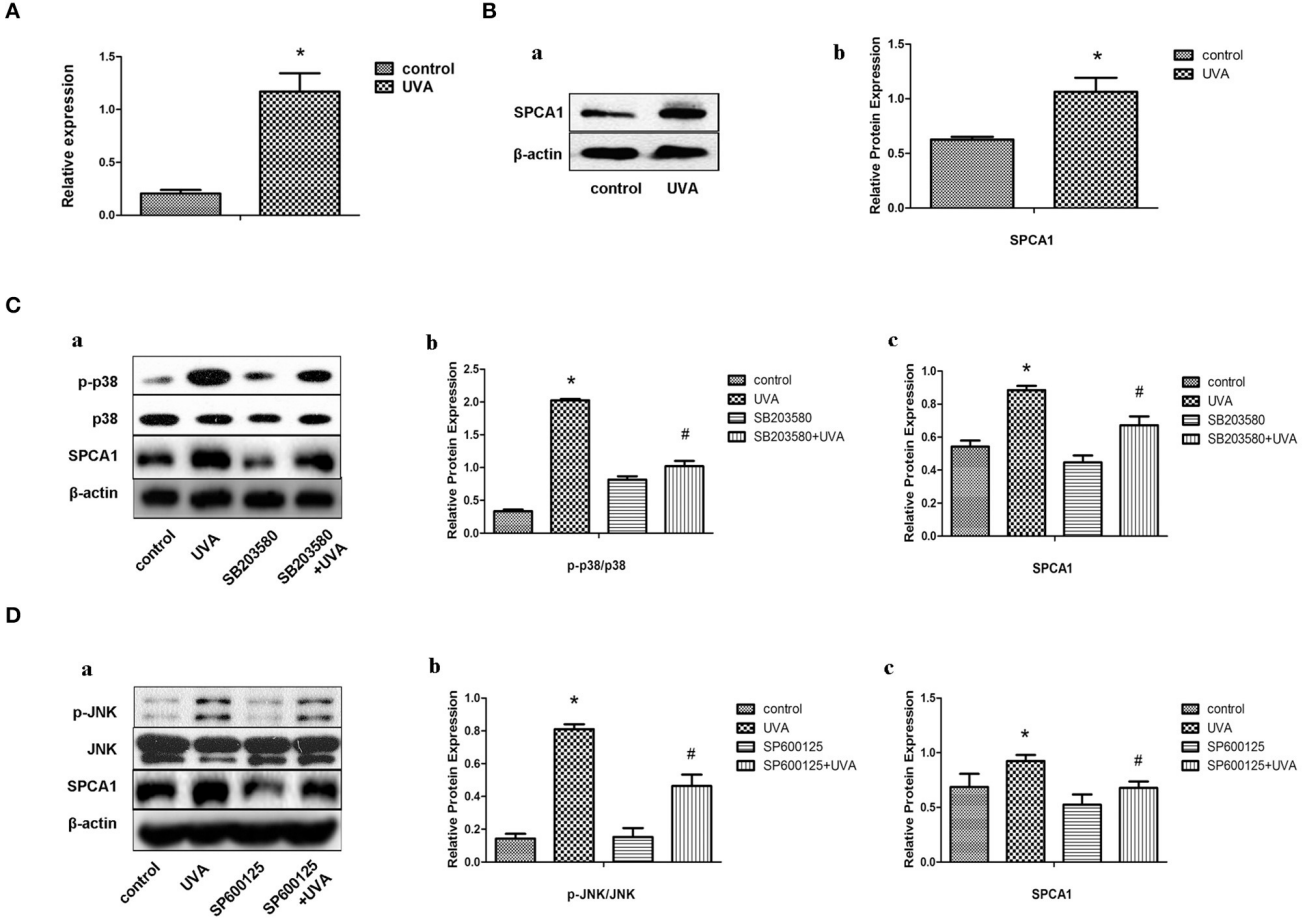
UVA irradiation increases the expression of SPCA1 by activating the MAPK pathway. **(A)** SPCA1 mRNA expression in control and UVA-irradiated HDFs, as determined by real-time PCR. Each sample was analyzed in triplicate. Data are shown as the mean of three independent experiments. *P < 0.05 vs. control. **(B)** (a) SPCA1 protein expression in control and UVA-irradiated HDFs, as determined by Western blot analysis. Images are representative of three independent experiments. (b) Bar graphs show quantitative analysis of scanning densitometric values of SPCA1 as ratios to β-actin, which was used as a loading control. Data are representative of three independent experiments. *P < 0.05 vs. control. **(C,D)** Effects of SB203580 and SP600125 (inhibiter of p38 and JNK) on UVA-induced SPCA1 protein expression and phosphorylation of p38 and JNK as determined by Western blot analysis. Control and UVA-irradiated HDFs were treated with or without 10 μM SB203580 and SP600125, respectively. Images are representative of three independent experiments. (b,c) Bar graphs show quantitative analysis of scanning densitometric values of SPCA1 as ratios to β-actin and phospho-JNK, p38 proteins as ratios to their total JNK, and p38, respectively. Data are representative of three independent experiments. *P < 0.05 vs. control; ^#^*P* < 0.05 vs. UVA.

Primary HDFs were irradiated with 10 J/cm^2^ UVA/day for 3 days. Both the mRNA and protein levels of SPCA1 were remarkably increased in HDFs after UVA irradiation (Figures 1A,B). Then, we investigated the possible signaling pathway regulating SPCA1 expression. UVA irradiation enhanced phosphorylation of P38 and JNK, while inhibitors of MAPK (SB203580 and SP600125) suppressed this phosphorylation, as well as the SPCA1 expression induced by UVA irradiation (Figures 1C,D). These results indicate that UVA irradiation increases the expression of SPCA1 by activating the MAPK pathway.

### MAPK Transcriptionally Regulates SPCA1 via Activating c-Jun

**Figure 2:**
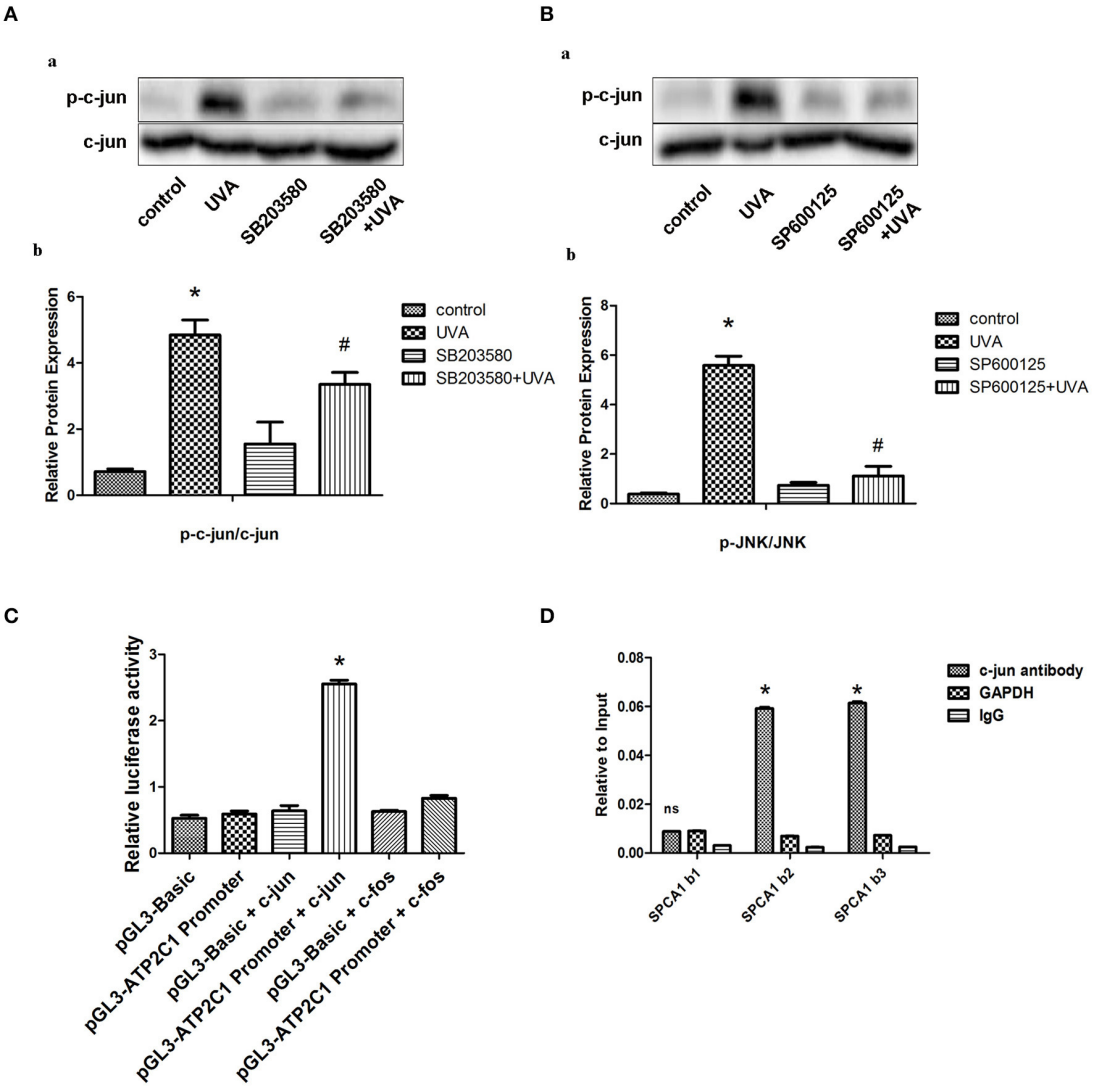
MAPK transcriptionally regulates SPCA1 via activating c-jun. **(A,B)** Western blot images and quantitative analysis show effects of SB203580 and SP600125 on UVA-induced phosphorylation of c-jun. Images are representative of three independent experiments. *P < 0.05 vs. control; ^#^*P* < 0.05 vs. SB203580 + UVA or SP600125 + UVA. The treatment of SB203580 and SP600125 is described above. **(C)** Luciferase reporter assay data, showing the activity of SPCA1 promoter. Cells were transfected with the following plasmids: c-jun, c-jun-cDNA-expressing vector; c-fos, c-fos-cDNA-expressing vector; ATP2C1, reporter plasmid containing SPCA1 promoter. Experiments were performed in triplicate. *P < 0.05 vs. pGL3-ATP2C1 promoter. **(D)** Chromatin immunoprecipitation data from HDFs incubated with either anti-SPCA1 antibody or non-specific control IgG, showing the amplification of each of the four predicted c-jun-binding sites within the SPCA1 promoter (termed SPCA1 b1, b2, and b3). Experiments were performed in triplicate.*P < 0.05 vs. IgG; ^ns^*P* < 0.05 vs. IgG.

AP-1 is a classical transcription factor activated by MAPK. Phosphorylation of c-jun was increased after UVA irradiation, which could be blocked by MAPK inhibitors (Figures 2A,B). We predicted three high score binding sites of c-jun on the SPCA1 promoter through JASPAR bioinformatics software. Basic information about these three binding sites is presented in the Supporting Information (Supplementary Table 1). We cloned the promoter of SPCA1 (−2,000 to 0 bp) and generated a SPCA1 promoter luciferase reporter and cotransfected to HEK293T cell with p6600 MSCV-IP N-HA only JUN or pLX304-FOS-V5 (c-jun and c-fos expression plasmid). The dual-luciferase reporter assay revealed that c-jun markedly enhanced activity of SPCA1 promoter luciferase reporter, but c-fos (another component of AP-1) had no effect on SPCA1 promoter activity (Figure 2C). Furthermore, ChIP assay was applied to determine whether c-jun was bound to these sequences directly. The primers for amplifying these predicted binding sites in ChIP assays is shown in Supplementary Table 2. The results showed that the sequences at the predicted binding sites 2 and 3 were amplified to a greater extent following immunoprecipitation with an anti-c-jun antibody than with the non-specific IgG control (Figure 2D). These data suggest that AP-1 might bind directly to the predicted binding sites 2 and/or 3 of the SPCA1 promoter and regulates its transcription level. This indicated that c-jun might bind to the predicted sequence.

### SPCA1 siRNA Exacerbates UVA-Induced Senescence and Phosphorylation of MAPK

**Figure 3:**
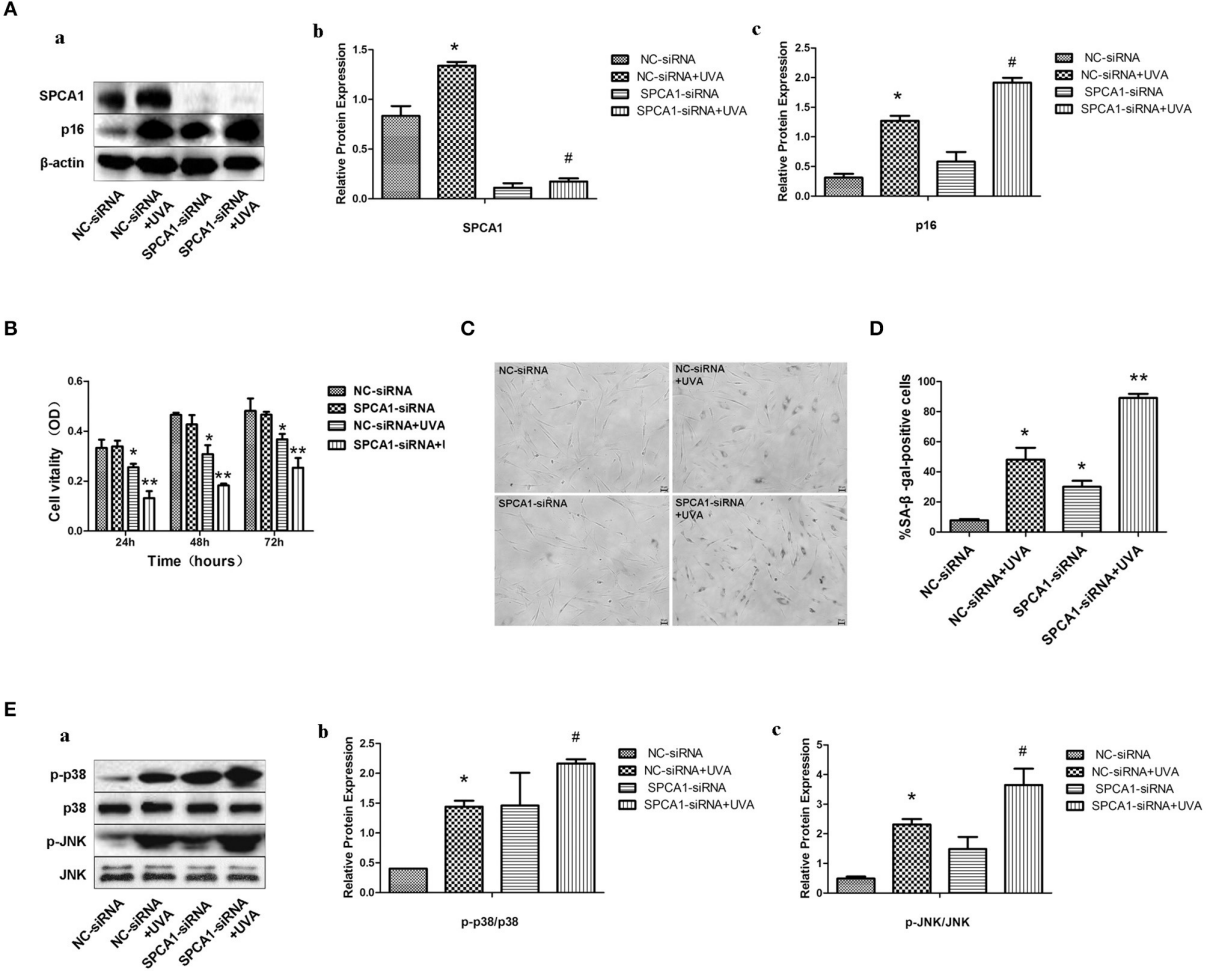
SPCA1 siRNA exacerbates UVA-induced senescence and phosphorylation of MAPK. **(A)** Western blot images and quantitative analysis show SPCA1 and p16 protein expression in control and UVA-irradiated HDFs, with or without SPCA1 siRNA transfection. *P < 0.05 vs. NC-siRNA; ^#^*P* < 0.05 vs. NC-siRNA + UVA. Data are representative of three independent experiments. **(B)** Cell vitality determined by MTT analysis under indicated conditions. *P < 0.05 vs. NC-siRNA; **P < 0.05 vs. NC-siRNA + UVA. **(C,D)** Senescence-associated β-galactosidase (SA-β-gal) activity of cells under the indicated conditions. Representative images are shown (scale bar = 200 μm). The percentages of SA-β-gal-positive cells under each condition are presented as the mean ± standard deviation of three independent experiments. *P < 0.05 vs. NC-siRNA; **P < 0.05 vs. NC-siRNA + UVA. **(E)** Western blot images and quantitative analysis show phosphorylation of p38 and JNK in control and UVA-irradiated HDFs, with or without SPCA1 siRNA transfection. *P < 0.05 vs. NC-siRNA; ^#^*P* < 0.05 vs. NC-siRNA + UVA. Data are representative of three independent experiments.

To identify the role of SPCA1 in UVA-induced senescence in HDFs, SPCA1 siRNA was transfected to decrease its expression. SA-β-gal activity, and the expression of p16 (hallmark of cellular senescence), were measured to evaluate cellular senescence. Downregulation of SPCA1 exacerbated the increase of SA-β-gal-positive cells and the expression of p16 induced by UVA irradiation (Figures 3A,C,D); 24, 48, and 72 h after UVA radiation, SPCA1 and NS siRNA-transfected HDFs both decreased in cell viability, but the cell viability dropped severely in SPCA1 siRNA-transfected groups (Figure 3B). Downregulation of SPCA1 also promoted the phosphorylation of MAPK caused by UVA (Figure 3E). Thus, we demonstrated that downregulating SPCA1 exacerbates UVA-induced senescence and MAPK activation.

### SPCA1 cDNA Attenuates UVA-Induced Senescence and Phosphorylation of MAPK

**Figure 4:**
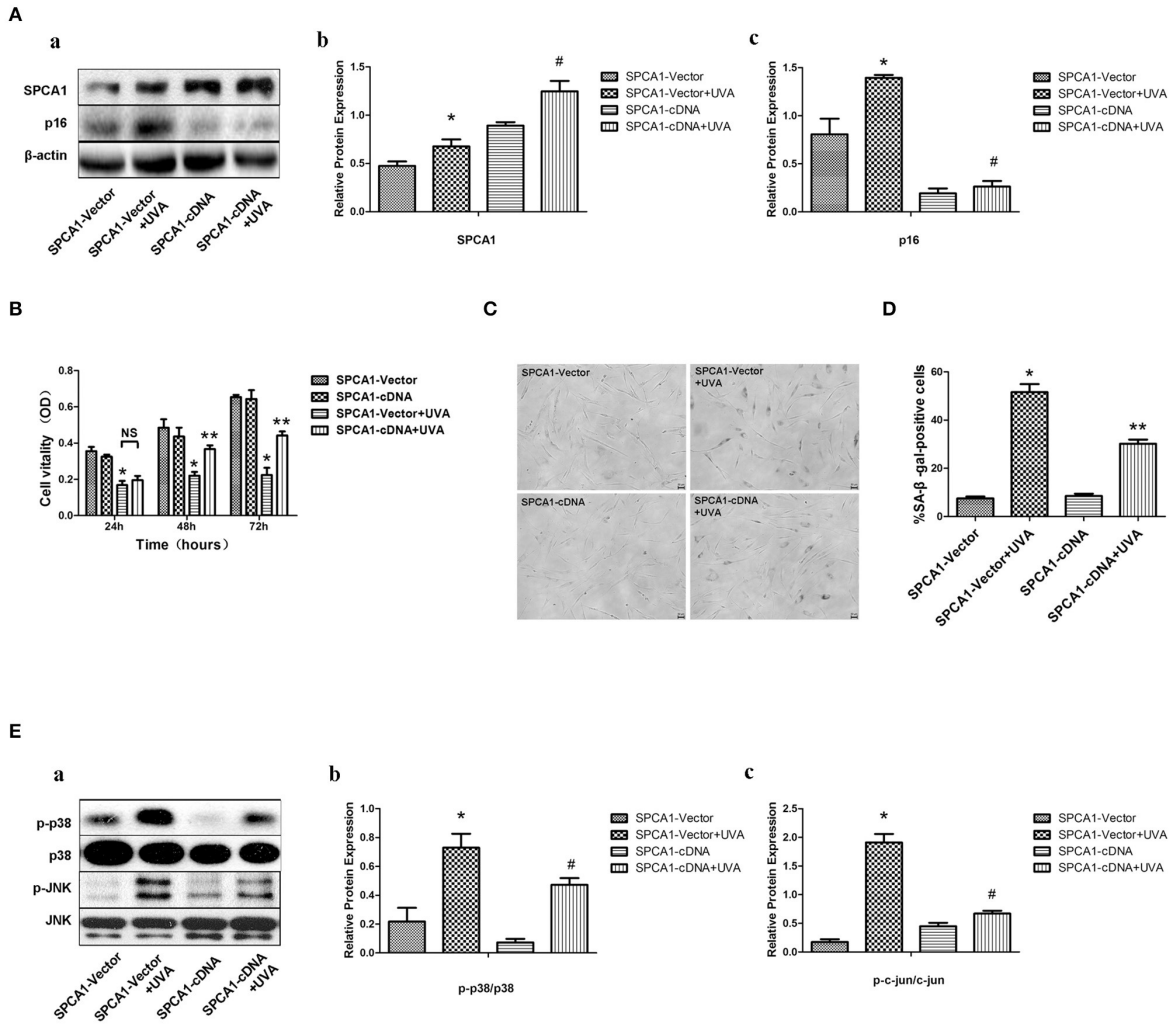
SPCA1 cDNA attenuates UVA-induced senescence and phosphorylation of MAPK. **(A)** Western blot images and quantitative analysis show SPCA1 and p16 protein expression in control and UVA-irradiated HDFs, with or withoutSPCA1 cDNA transfection, *P < 0.05 vs. SPCA1-vector; ^#^*P* < 0.05 vs. SPCA1-vector+UVA. Data are representative of three independent experiments. **(B)** Cell vitality determined by MTT analysis under indicated conditions. *P < 0.05, SPCA1-vector + UVA vs. SPCA1-vector; **P < 0.05, SPCA1-cDNA+UVA vs. SPCA1-vector+UVA. **(C,D)** Senescence-associated β-galactosidase (SA-β-gal) activity of cells under the indicated conditions. Representative images are shown (scale bar = 200 μm). The percentages of SA-β-gal-positive cells under each condition are presented as the mean ± standard deviation of three independent experiments. *P < 0.05 vs. SPCA1-vector; **P < 0.05 vs. SPCA1-vector+UVA. **(E)** Western blot images and quantitative analysis show phosphorylation of p38 and JNK in control and UVA-irradiated HDFs, with or without SPCA1 cDNA transfection. *P < 0.05 vs. SPCA1-vector; ^#^*P* < 0.05 vs. SPCA1-vector + UVA. Data are representative of three independent experiments.

SPCA1 cDNA was nucleofected into HDFs to further investigate the role of SPCA1 in UVA-induced senescence. Upregulation of SPCA1 partially reversed the increased number of SA-β-gal-positive cells (Figures 4C,D), and p16 expression (Figure 4A), as well as phosphorylation of MAPK caused by UVA irradiation (Figure 4E). Simultaneously, the UVA-induced reduction of cell viability was partially reversed by SPCA1 cDNA (Figure 4B). These results indicate that overexpression of SPCA1 attenuates UVA-induced senescence and phosphorylation of MAPK in HDFs.

### SPCA1 Affects UVA-Induced ROS and MAPK Activation via Regulating [Ca^2+^]i

**Figure 5:**
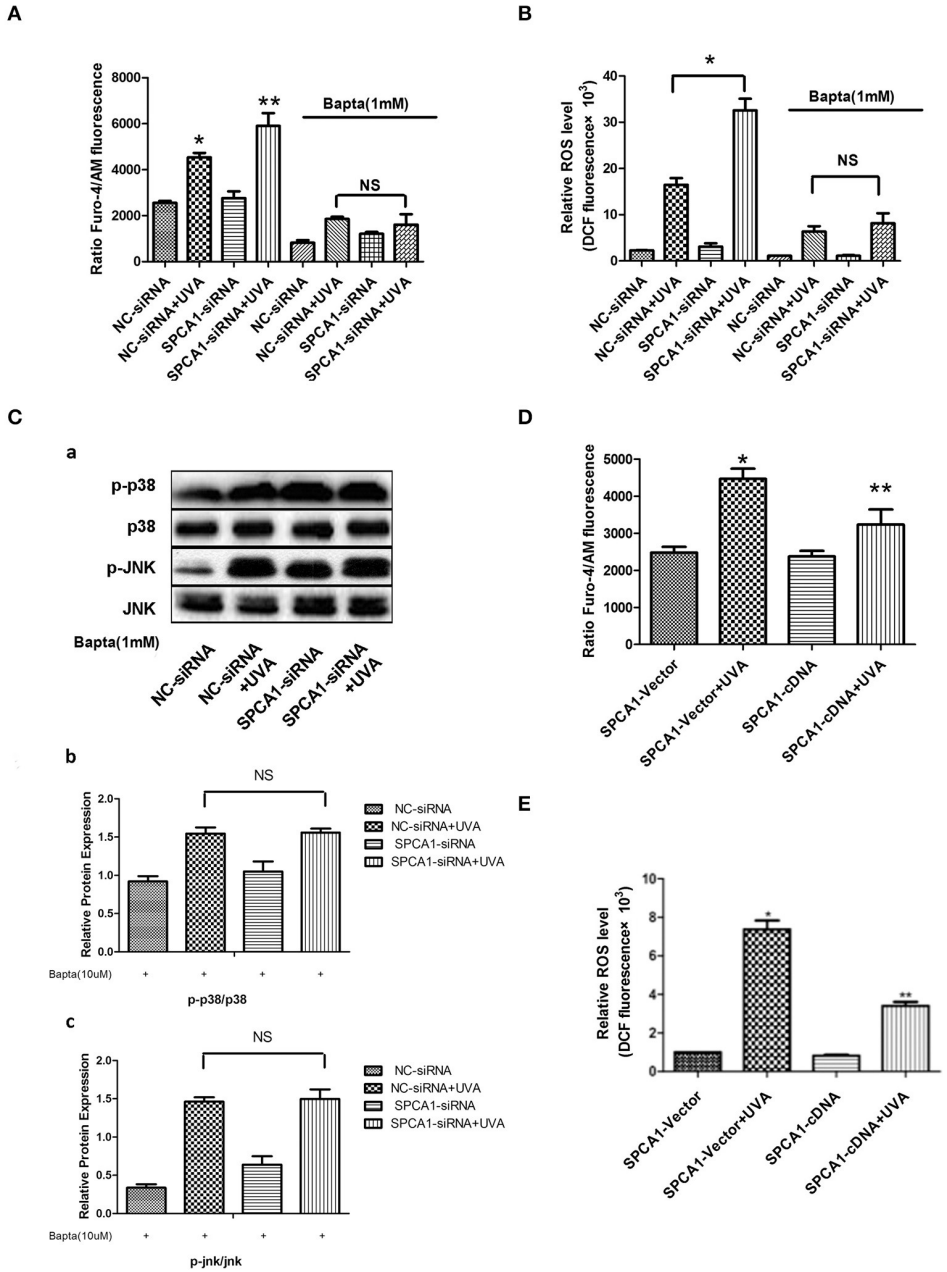
SPCA1 affects UVA-induced ROS and MAPK activation via regulating [Ca^2+^]i. **(A)** [Ca^2+^]i levels determined by Fluo-4/AM fluorescence under indicated conditions. Bapta (1 mM) abolish the effect of SPCA1 siRNA on [Ca^2+^]i levels in UVA-irradiated HDF. *P < 0.05 vs. NC-siRNA; **P < 0.05 vs. NC-siRNA + UVA; ^ns^*P* < 0.05. **(B)** ROS levels determined by dichlorofluorescin diacetate fluorescence under indicated conditions. Bapta (1 mM) abolish the effect of SPCA1 siRNA on ROS levels in UVA-irradiated HDF. *P < 0.05 vs. NC-siRNA; **P < 0.05 vs. NC-siRNA + UVA; ^ns^*P* < 0.05. **(C)** Western blot images and quantitative analysis show Bapta (1 mM) abolish the effect of SPCA1 siRNA on phosphorylation of p38 and JNK in UVA-irradiated HDF. ^ns^*P* < 0.05. Data are representative of three independent experiments. **(D,E)** [Ca^2+^]i and ROS level in control and UVA-irradiated HDFs, with or without SPCA1 cDNA transfection. *P < 0.05 vs. SPCA1-vector; **P < 0.05 vs. SPCA1-vector + UVA.

To investigate the possible mechanism of the effects of SPCA1 on UVA-induced senescence, we evaluated the levels of [Ca^2+^]i and ROS. Downregulating SPCA1 further elevated the increase of calcium, ROS, and MAPK activity induced by UVA. Moreover, compared with HDFs transfected with NC siRNA, UVA-irradiated HDFs transfected with SPCA1 siRNA have no difference in ROS level and phosphorylation of MAPK at the presence of BAPTA (Figures 5A–C). On the contrary, the elevations of intracellular calcium, ROS, and MAPK activity initiated by UVA were partially reversed by SPCA1 cDNA transfection (Figures 5D,E). Thus, our results demonstrate that SPCA1 influence UVA-induced ROS and MAPK activation by regulating [Ca^2+^]i.

## Discussion

In this study, we found that UVA irradiation promoted the expression of SPCA1 through activation of MAPK and its downstream transcription factor, c-jun, which directly binds to the SPCA1 promoter. Increased SPCA1 suppressed UVA-induced MAPK activity by lowering the [Ca^2+^]i and ROS levels, thereby alleviating cellular senescence caused by UVA. Thus, a novel negative feedback loop to maintain homeostasis in HDFs under UVA irradiation was discovered. This is the initial research concerning a role for SPCA1 and its regulatory mechanism in cellular senescence, suggesting that SPCA1 is involved in self-defense in UVA-induced senescence.

Oxidative stress and ROS are decisive factors of UVA-induced senescence and MAPK activation. Several reports in different areas congruously found that SPCA1 was associated with oxidative stress and ROS due to its capability of regulating [Ca^2+^]i; some refer to this as “Golgi stress” (Okunade et al., 2007; Shull et al., 2011). Keratinocytes derived from Hailey–Hailey disease patients, which lack one functional copy of the ATP2C1 gene, underwent oxidative stress, while ATP2C1 inactivation increased oxidative stress in cultured human keratinocytes (Cialfi et al., 2010, 2016). In addition, there is a correlation between SPCA1 and oxidative stress in ischemia/reperfusion and ischemic preconditioning of brain cells (Pavlikova et al., 2009). Similarly, our findings suggested that SPCA1 played a very important role in UVA-induced senescence by altering oxidative stress. Silencing SPCA1 exacerbated the increased [Ca^2+^]i induced by UVA irradiation, leading to higher ROS levels and MAPK activity and eventually aggravating cellar senescence in HDFs. In contrast, overexpression of SPCA1 yielded the opposite result and exhibited a protective effect on senescence. The effects of SPCA1 siRNA on ROS and MAPK activity could be abolished by a calcium chelator, BAPTA. This indicates that [Ca^2+^]i acted as a mediator between SPCA1 and oxidative stress. Nevertheless, the specific underlying mechanism involved in regulating ROS by [Ca^2+^]i in this situation requires further study. It has been shown that intracellular calcium could cause mitochondria Ca^2+^ overload (Li et al., 2013), induce a three-dimensional conformation change of the respiratory chain complexes (Brookes et al., 2004), increase metabolic rate (Brookes et al., 2004), activate cytoplasmic NADPH oxidases (NOXes) (Crosas-Molist and Fabregat, 2015; Gorlach et al., 2015), and all eventually increase ROS production. Some of the processes mentioned above may be involved in our case.

Because SPCA1 has a protective role in UVA-induced cellular senescence, we examined its upstream regulatory mechanism. Loss of one functional copy of the ATP2C1 gene causes low expression of SPCA1 and Hailey–Hailey disease, a human autosomal dominant skin disorder characterized by suprabasal acantholysis of keratinocytes (Hu et al., 2000; Sudbrak et al., 2000). Short wave increased expression of SPCA1 in middle cerebral artery occlusion (Fan et al., 2016a), ischemia/reperfusion depressed SPCA1 expression, and ischemic preconditioning could partially reverse this kind of depression in hippocampal cells (Lehotsky et al., 2009). Serotonin deficiency decreased the expression of SPCA1 mRNA in mammary epithelial cells (Laporta et al., 2014). However, these reports only provided the expression changes of SPCA1 in certain conditions, without exploring the underlying mechanism. Aside from these findings, little is known about the regulatory mechanism of SPCA1, especially in skin.

In our study, we not only discovered a new condition that modified SPCA1 expression:UVA irradiation but also revealed a new regulatory mechanism of SPCA1. Specifically, UVA-activated MAPK and its downstream transcription factor c-jun, which, in turn, directly bound to the SPCA1 promoter and up-regulated its expression.

MAPK activation plays a crucial role in UVA-induced senescence. For example, MAPK increases MMP1, MMP3, and MMP9 through activating AP-1, leading to collagen degradation (Wang et al., 2005; Kim et al., 2013). Alternately, MAPK can directly phosphorylate p16 and p53 and initiate senescence of skin cells, especially dermal fibroblasts (Bulavin et al., 1999; Singh et al., 2003). In our previous studies, we demonstrated that the MAPK pathway was involved in UVA-induced senescence and apoptosis (Xie et al., 2013; Wang et al., 2015). In agreement with this finding, our present research also showed the activation of MAPK and c-jun, under UVA irradiation. Furthermore, we identified SPCA1 as a new target gene regulated by MAPK, in UVA-induced senescence in HDFs by using MAPK inhibitors.

MAPK activates c-jun by phosphorylation, and the latter binds to specific sequences called the tetradeconylphorbol-13-acetate response elements (TREs) in the promoters of AP-1-inducible genes, contributing to transcriptional activation or repression of target genes (Angel and Karin, 1991; Whitmarsh and Davis, 1996). We also found that UVA phosphorylated c-jun via activation of MAPK by using selective inhibitors. Using bioinformatic software (JASPAR), we predicted three TREs on the promoter of SPCA1. Dual-luciferase reporter and ChIP assays located the precise functional domain on the SPCA1 promoter that bound c-jun. To our knowledge, this regulatory mechanism of SPCA1 has not been reported previously and might provide a new direction for research concerning the regulation of SPCA1 in different circumstances.

Negative feedback is a core mechanism to maintain homeostasis and cope with stress in the human body. Examples of this are numerous, from the baroreflex in blood pressure to the regulation of hormone secretion. Overall, our research suggests that SPCA1 might exert a protective effect on UVA-induced senescence in HDFs through negative feedback of MAPK. Activation of MAPK/c-jun triggered by UVA could transcriptionally upregulate SPCA1. In turn, increased SPCA1 brings down the [Ca^2+^]i, probably through pumping Ca^2+^ into the Golgi apparatus. This reduces ROS levels, eventually decreasing MAPK activity and easing UVA-induced senescence. Therefore, this negative feedback loop could partially break the signaling cascade of MAPK, alleviate damage caused by UVA, maintain cellular homeostasis, and prevent cells from senescence.

## Data Availability Statement

The original contributions presented in the study are included in the article/Supplementary Material, further inquiries can be directed to the corresponding author/s.

## Ethics Statement

Before harvesting primary HDFs, written informed consent was obtained from legal guardians of donors in accordance with a protocol approved by the Clinical Research Ethics Committee at the Xiang Ya Hospital of Central South University in Changsha, China.

## Author Contributions

DJ and ZD performed the experiments, analyzed the data, and wrote the manuscript. MD and PL performed the experiments. YY and HX analyzed the data. JL and XX discussed the analyses, interpretation, and presentation and edited the manuscript. All authors contributed to the article and approved the submitted version.

## Conflict of Interest

The authors declare that the research was conducted in the absence of any commercial or financial relationships that could be construed as a potential conflict of interest.

## References

[B1] Angel P. Karin M. (1991). The role of Jun, Fos and the AP-1 complex in cell-proliferation and transformation. Bioch. Biophys. Acta 1072, 129–157. 10.1016/0304-419X(91)90011-9 1751545

[B2] Bosch R. Philips N. Suarez-Perez J. A. Juarranz A. Devmurari A. Chalensouk-Khaosaat J.. (2015). Mechanisms of photoaging and cutaneous photocarcinogenesis, and photoprotective strategies with phytochemicals. Antioxidants 4, 248–268. 10.3390/antiox4020248 26783703 PMC4665475

[B3] Brookes P. S. Yoon Y. Robotham J. L. Anders M. W. Sheu S. S. (2004). Calcium, A. T. P., and ROS: a mitochondrial love-hate triangle. Am. J. Physiol. Cell Physiol. 287, C817–C833. 10.1152/ajpcell.00139.2004 15355853

[B4] Bulavin D. V. Saito S. Hollander M. C. Sakaguchi K. Anderson C. W. Appella E.. (1999). Phosphorylation of human p53 by p38 kinase coordinates N-terminal phosphorylation and apoptosis in response to UV radiation. EMBO J. 18, 6845–6854. 10.1093/emboj/18.23.6845 10581258 PMC1171747

[B5] Chaiprasongsuk A. Lohakul J. Soontrapa K. Sampattavanich S. Akarasereenont P. Panich U. (2017). Activation of Nrf2 reduces UVA-Mediated MMP-1 upregulation via MAPK/AP-1 signaling cascades: the photoprotective effects of sulforaphane and hispidulin. J. Pharmacol. Exp. Ther. 360, 388–398. 10.1124/jpet.116.238048 28011874 PMC5325073

[B6] Cialfi S. Le Pera L. De Blasio C. Mariano G. Palermo R. Zonfrilli A.. (2016). The loss of ATP2C1 impairs the DNA damage response and induces altered skin homeostasis: consequences for epidermal biology in Hailey-Hailey disease. Sci. Rep. 6:31567. 10.1038/srep31567 27528123 PMC4985699

[B7] Cialfi S. Oliviero C. Ceccarelli S. Marchese C. Barbieri L. Biolcati G.. (2010). Complex multipathways alterations and oxidative stress are associated with Hailey-Hailey disease. Br. J. Dermatol. 162, 518–526. 10.1111/j.1365-2133.2009.09500.x 19903178

[B8] Crosas-Molist E. Fabregat I. (2015). Role of NADPH oxidases in the redox biology of liver fibrosis. Redox Biol. 6, 106–111. 10.1016/j.redox.2015.07.005 26204504 PMC4804101

[B9] Fan Y. Zhang C. Li T. Peng W. Yin J. Li X.. (2016a). A new approach of short wave protection against middle cerebral artery occlusion/reperfusion injury via attenuation of golgi apparatus stress by inhibition of downregulation of secretory pathway Ca(2+)-ATPase isoform 1 in rats. J. Stroke Cerebrovasc. Dis. 25, 1813–1822. 10.1016/j.jstrokecerebrovasdis.2016.03.033 27133772

[B10] Fan Y. Zhang C. Peng W. Li T. Yin J. Kong Y.. (2016b). Secretory pathway Ca(2+)-ATPase isoform 1 knockdown promotes Golgi apparatus stress injury in a mouse model of focal cerebral ischemia-reperfusion: *in vivo* and *in vitro* study. Brain Res. 1642, 189–196. 10.1016/j.brainres.2016.03.049 27038757

[B11] Fisher G. J. Kang S. Varani J. Bata-Csorgo Z. Wan Y. Datta S.. (2002). Mechanisms of photoaging and chronological skin aging. Arch. Dermatol. 138, 1462–1470. 10.1001/archderm.138.11.1462 12437452

[B12] Gorlach A. Bertram K. Hudecova S. Krizanova O. (2015). Calcium and ROS: a mutual interplay. Redox Biol. 6, 260–271. 10.1016/j.redox.2015.08.010 26296072 PMC4556774

[B13] Grice D. M. Vetter I. Faddy H. M. Kenny P. A. Roberts-Thomson S. J. Monteith G. R. (2010). Golgi calcium pump secretory pathway calcium ATPase 1 (SPCA1) is a key regulator of insulin-like growth factor receptor (IGF1R) processing in the basal-like breast cancer cell line MDA-MB-231. J. Biol. Chem. 285, 37458–37466. 10.1074/jbc.M110.163329 20837466 PMC2988351

[B14] Hu Z. Bonifas J. M. Beech J. Bench G. Shigihara T. Ogawa H.. (2000). Mutations in ATP2C1, encoding a calcium pump, cause Hailey-Hailey disease. Nat. Genet. 24, 61–65. 10.1038/71701 10615129

[B15] Kim J. M. Noh E. M. Kwon K. B. Hwang B. M. Hwang J. K. You Y. O.. (2013). Dihydroavenanthramide D prevents UV-irradiated generation of reactive oxygen species and expression of matrix metalloproteinase-1 and−3 in human dermal fibroblasts. Exp. Dermatolo. 22, 759–761. 10.1111/exd.12243 24103002 PMC4251632

[B16] Krutmann J. (2001). The role of UVA rays in skin aging. Eur. J. Dermatol. 11, 170–171. 11275823

[B17] Laporta J. Keil K. P. Vezina C. M. Hernandez L. L. (2014). Peripheral serotonin regulates maternal calcium trafficking in mammary epithelial cells during lactation in mice. PLoS ONE. 9:e110190. 10.1371/journal.pone.0110190 25299122 PMC4192539

[B18] Lehotsky J. Racay P. Pavlikova M. Tatarkova Z. Urban P. Chomova M.. (2009). Cross-talk of intracellular calcium stores in the response to neuronal ischemia and ischemic tolerance. Gen. Physiol. Biophys. 28:F104–F114. 20093720

[B19] Li X. Fang P. Mai J. Choi E. T. Wang H. Yang X. F. (2013). Targeting mitochondrial reactive oxygen species as novel therapy for inflammatory diseases and cancers. J. Hematol. Oncol. 25, 6–19. 10.1186/1756-8722-6-19 23442817 PMC3599349

[B20] Micaroni M. Giacchetti G. Plebani R. Xiao G. G. Federici L. (2016). ATP2C1 gene mutations in Hailey-Hailey disease and possible roles of SPCA1 isoforms in membrane trafficking. Cell Death Dis. 7:e2259. 10.1038/cddis.2016.147 27277681 PMC5143377

[B21] Micaroni M. Perinetti G. Berrie C. P. Mironov A. A. (2010). The SPCA1 Ca^2+^ pump and intracellular membrane trafficking. Traffic 11, 1315–1333. 10.1111/j.1600-0854.2010.01096.x 20604898

[B22] Missiaen L. Dode L. Vanoevelen J. Raeymaekers L. Wuytack F. (2007). Calcium in the Golgi apparatus. Cell Calcium 41, 405–416. 10.1016/j.ceca.2006.11.001 17140658

[B23] Missiaen L. Raeymaekers L. Dode L. Vanoevelen J. Van Baelen K. Parys J. B.. (2004). SPCA1 pumps and Hailey-Hailey disease. Biochem. Biophys. Res. Commun. 322, 1204–1213. 10.1016/j.bbrc.2004.07.128 15336968

[B24] Nellen R. G. Steijlen P. M. van Steensel M. A. Vreeburg M. European Professional C. Frank J.. (2017). Mendelian disorders of cornification caused by defects in intracellular calcium pumps: mutation update and database for variants in ATP2A2 and ATP2C1 associated with darier disease and hailey-hailey disease. Hum. Mutation 38, 343–356. 10.1002/humu.23164 28035777

[B25] Okunade G. W. Miller M. L. Azhar M. Andringa A. Sanford L. P. Doetschman T.. (2007). Loss of the Atp2c1 secretory pathway Ca(2+)-ATPase (SPCA1) in mice causes Golgi stress, apoptosis, and midgestational death in homozygous embryos and squamous cell tumors in adult heterozygotes. J. Biol. Chem. 282, 26517–26527. 10.1074/jbc.M703029200 17597066

[B26] Pavlikova M. Tatarkova Z. Sivonova M. Kaplan P. Krizanova O. Lehotsky J. (2009). Alterations induced by ischemic preconditioning on secretory pathways Ca^2+^-ATPase (SPCA) gene expression and oxidative damage after global cerebral ischemia/reperfusion in rats. Cell. Mol. Neurobiol. 29, 909–916. 10.1007/s10571-009-9374-6 19288187 PMC11506051

[B27] Praitis V. Simske J. Kniss S. Mandt R. Imlay L. Feddersen C.. (2013). The secretory pathway calcium ATPase PMR-1/SPCA1 has essential roles in cell migration during *Caenorhabditis elegans* embryonic development. PLoS Genet. 9:e1003506. 10.1371/journal.pgen.1003506 23696750 PMC3656159

[B28] Reinhardt T. A. Lippolis J. D. (2009). Mammary gland involution is associated with rapid down regulation of major mammary Ca^2+^-ATPases. Biochem. Biophys. Res. Commun. 378, 99–102. 10.1016/j.bbrc.2008.11.004 19000904

[B29] Rinnerthaler M. Bischof J. Streubel M. K. Trost A. Richter K. (2015). Oxidative stress in aging human skin. Biomolecules 5, 545–589. 10.3390/biom5020545 25906193 PMC4496685

[B30] Sepulveda M. R. Marcos D. Berrocal M. Raeymaekers L. Mata A. M. Wuytack F. (2008). Activity and localization of the secretory pathway Ca^2+^-ATPase isoform 1 (SPCA1) in different areas of the mouse brain during postnatal development. Mol. Cell. Neurosci. 38, 461–473. 10.1016/j.mcn.2008.02.012 18599310

[B31] Sepulveda M. R. Vanoevelen J. Raeymaekers L. Mata A. M. Wuytack F. (2009). Silencing the SPCA1 (secretory pathway Ca^2+^-ATPase isoform 1) impairs Ca^2+^ homeostasis in the Golgi and disturbs neural polarity. J. Neurosci. 29, 12174–12182. 10.1523/JNEUROSCI.2014-09.2009 19793975 PMC6666140

[B32] Shull G. E. Miller M. L. Prasad V. (2011). Secretory pathway stress responses as possible mechanisms of disease involving Golgi Ca^2+^ pump dysfunction. BioFactors 37, 150–158. 10.1002/biof.141 21674634 PMC3338190

[B33] Singh S. Powell D. W. Rane M. J. Millard T. H. Trent J. O. Pierce W. M.. (2003). Identification of the p16-Arc subunit of the Arp 2/3 complex as a substrate of MAPK-activated protein kinase 2 by proteomic analysis. J. Biol. Chem. 278, 36410–36417. 10.1074/jbc.M306428200 12829704

[B34] Sudbrak R. Brown J. Dobson-Stone C. Carter S. Ramser J. White J.. (2000). Hailey-Hailey disease is caused by mutations in ATP2C1 encoding a novel Ca(2+) pump. Hum. Mol. Genet. 9, 1131–1140. 10.1093/hmg/9.7.1131 10767338

[B35] von Blume J. Alleaume A. M. Cantero-Recasens G. Curwin A. Carreras-Sureda A. Zimmermann T.. (2011). ADF/cofilin regulates secretory cargo sorting at the TGN via the Ca^2+^ ATPase SPCA1. Dev. Cell 20, 652–662. 10.1016/j.devcel.2011.03.014 21571222

[B36] von Blume J. Alleaume A. M. Kienzle C. Carreras-Sureda A. Valverde M. Malhotra V. (2012). Cab45 is required for Ca(2+)-dependent secretory cargo sorting at the trans-Golgi network. J. Cell Biol. 199, 1057–1066. 10.1083/jcb.201207180 23266954 PMC3529532

[B37] Wang B. Xie H. F. Li W. Z. Huang Y. X. Shi W. Jian D.. (2015). Asymmetrical dimethylarginine promotes the senescence of human skin fibroblasts via the activation of a reactive oxygen species-p38 MAPK-microRNA-138 pathway. J. Dermatol. Sci. 78, 161–164. 10.1016/j.jdermsci.2015.02.019 25818871

[B38] Wang X. Bi Z. Chu W. Wan Y. (2005). IL-1 receptor antagonist attenuates MAP kinase/AP-1 activation and MMP1 expression in UVA-irradiated human fibroblasts induced by culture medium from UVB-irradiated human skin keratinocytes. Int. J. Mol. Med. 16, 1117–1124. 10.3892/ijmm.16.6.1117 16273295

[B39] Whitmarsh A. J. Davis R. J. (1996). Transcription factor AP-1 regulation by mitogen-activated protein kinase signal transduction pathways. J. Mol. Med. 74, 589–607. 10.1007/s001090050063 8912180

[B40] Xie H. Liu F. Liu L. Dan J. Luo Y. Yi Y.. (2013). Protective role of AQP3 in UVA-induced NHSFs apoptosis via Bcl2 up-regulation. Arch. Dermatol. Res. 305, 397–406. 10.1007/s00403-013-1324-y 23463292

[B41] Yaar M. Gilchrest B. A. (2007). Photoageing: mechanism, prevention and therapy. Br. J. Dermatol. 157, 874–887. 10.1111/j.1365-2133.2007.08108.x 17711532

[B42] Zheng J. Lai W. Zhu G. Wan M. Chen J. Tai Y.. (2013). 10-Hydroxy-2-decenoic acid prevents ultraviolet A-induced damage and matrix metalloproteinases expression in human dermal fibroblasts. J. Eur. Acad. Dermatol. Venereol. 27, 1269–1277. 10.1111/j.1468-3083.2012.04707.x 23030720

