# Supplementary material for: A Negative Feedback Loop in Ultraviolet A-Induced Senescence in Human Dermal Fibroblasts Formed by SPCA1 and MAPK
Source: Front Cell Dev Biol. 2021 Jun 22;8:597993. doi: 10.3389/fcell.2020.597993 (PMC8259626; doi:10.3389/fcell.2020.597993)

Supplementary Information

Supplementary table 1

Basic information of three binding sites

Model ID	Model name	Score	Relative score	Start	End	Strand	predicted site sequence
MA0488.1	JUN	4.043	0.831752690982302	1250	1262	1	GAAGTGACCAAAT
MA0488.1	JUN	7.699	0.87157054435518	1393	1405	1	TTTATGATTTCAT
MA0488.1	JUN	13.721	0.937156735003832	1747	1759	1	AGAGTGAGGTAAT

Supplementary table 2

The primers for amplifying predicted binding sites in ChIP assays

binding site 1	Forward:5'- CTACATGACAGCCCCTCACA -3'<br>Reverse: 5'- CTGGTTCCTTTGATGGAAAC-3'
binding site 2	Forward:5'- AAAATATTGAACTTTCAAAT -3'<br>Reverse: 5'- GGTCTGTGGTCTTAGAGGAC -3'
binding site 3	Forward:5'- GAGCTCCTTGATAACAATGA -3'<br>Reverse: 5'- CTTCCAAAAAAATTTAACAA -3'

Materials and Methods

immunohistochemistry staining

Eight-week-old female FVB mice were obtained from the National Key Laboratory of Genetics (Changsha, Hunan, China).Animal experiments were approved by the Animal Research Committee at the Xiang Ya Hospital of Central South University. The dorsal skin area of mice was shaved before and during experiments. Mice were irradiated 3 times/week for 12 weeks with 20 J/cm2 doses under a Philips UVA lamp placed 20 cm away (emission

spectrum: 320–400 nm). Mice were sacrificed by cervical dislocation under chloral hydrate anesthesia at the end of experiments.

Matched sun-exposed (facial) and non-sun-exposed (abdominal) human skin specimens were taken from the same patient. For histological analyses, mice central dorsal skin specimens and human skin specimens were fixed in 4% paraformaldehyde and sectioned after paraffin embedding. Tissue immunohistochemistry staining was performed using anti-SPCA1 primary antibodies (1:100 dilution;Sangon Biotech), and a biotinylated rabbit anti-goat IgG secondary antibody(1:200 dilution； Beyotime). Detection was performed using a DAB Horseradish Peroxidase Color Development kit (Beyotime), according to the manufacturer’s protocol. Photographs of 3 randomly-chosen fields in each section were taken under a microscope(Magnification: 400x fold). Representative Photographs were presented.

Results

Expression of SPCA1 increases in sun-exposed human skin and UVA-irradiated mouse skin

Immunohistochemistry was performed on sun-exposed or non-sun-exposed human skin specimens from the same individuals, and dorsal skin of mice with or without UVA irradiation. Positive staining, which represents SPCA1 in sun-exposed human skin and UVA-irradiated mice skin, was increased significantly (SFig. 1).These *in vivo* results were in agreement with the changes of SPCA1 expression in HDFs.

Figure Legends

Supplementary Figure 1. Expression of SPCA1 increases in sun-exposed human skin and UVA-irradiated mouse skin. Representative immunohistochemical staining images of dorsal skin of mice with or without UVA irradiation (A, B) and matched human sun-exposed skin specimens from within the same individual(C, D). Sections were imaged using primary

antibodies raised against SPCA1 (Magnification: 400x fold).

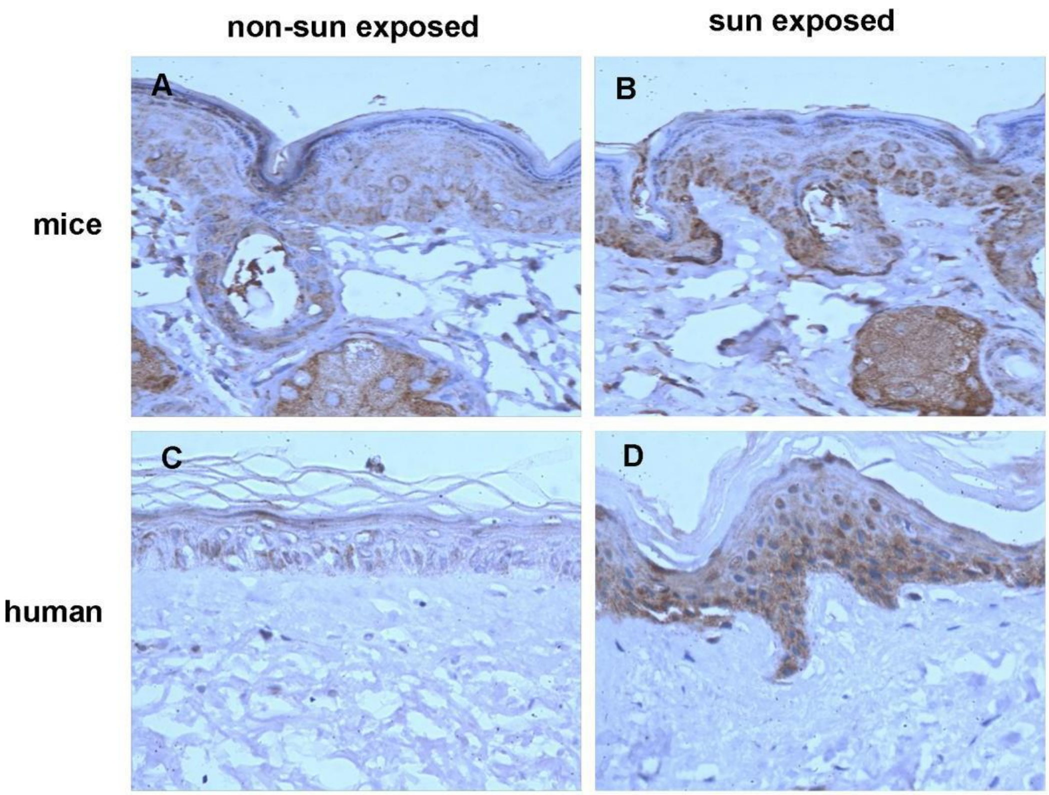

Supplementary Figure2. A negative feedback loop in ultraviolet A-induced senescence in human dermal fibroblasts formed by SPCA1 and MAPK activation of MAPK/c-jun triggered by UVA transcriptionally up regulated SPCA1. In turn, the increased SPCA1 lowered the intracellular Ca^{2+} level, leading to a reduction of ROS, eventually decreasing MAPK activity and diminishing UVA-induced senescence.

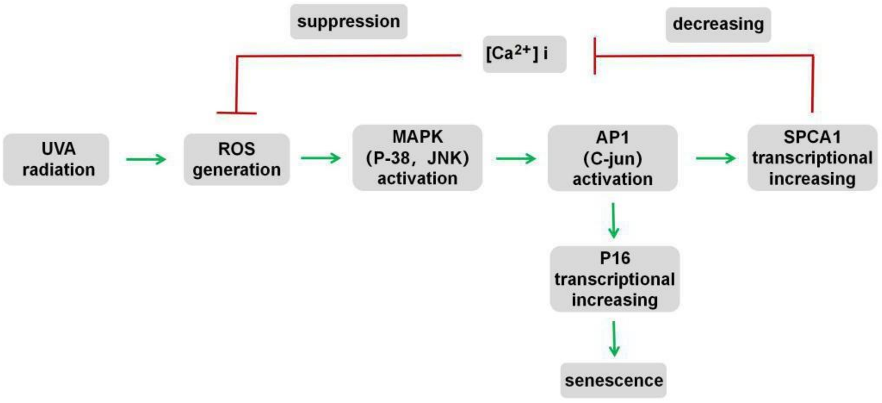

Supplement: Supplementary file 1 [file Data_Sheet_1.pdf]
